# Supplementary material for: Noncollinear harmonic spectroscopy reveals crossover of strong-field effects
Source: Nat Commun. 2025 Aug 18;16:7660. doi: 10.1038/s41467-025-62746-2 (PMC12361368; doi:10.1038/s41467-025-62746-2)
Supplement: Supplementary file 1 — Supplementary Information [file 41467_2025_62746_MOESM1_ESM.pdf]

Supplementary Information (SI) for

Noncollinear Harmonic Spectroscopy Reveals Crossover of Strong-Field Effects

Jicai Zhang[1*], Xiulan Liu[2,3], Tien-Dat Tran[1], Wenqi Xu[1], Wenhao Yu[1], Chong Zhang[1], Ziwen Wang[1], Lei Geng[2], Jianing Zhang[2], Liang-You Peng[2,3,4*], Stanislav Yu. Kruchinin[5*], Tran Trung Luu[1*]

[1]Department of Physics, The University of Hong Kong; Pok Fu Lam Rd, Hong Kong SAR, China

[2]State Key Laboratory for Mesoscopic Physics and Frontiers Science Center for Nano-optoelectronics, School of Physics, Peking University, Beijing, 100871, China

[3]Beijing Academy of Quantum Information Sciences, Beijing, 100193, China

[4]Collaborative Innovation Center of Extreme Optics, Shanxi University, Taiyuan, 030006 China

[5]Microsoft Austria, Am Europlatz 3, 1120 Vienna, Austria

*Corresponding author.

E-Mail: jczhang@hku.hk; liangyou.peng@pku.edu.cn; stanislav.kruchinin@microsoft.com; ttluu@hku.hk

Content

1 Experimental Methods, Data Processing, and Sample Properties ... 3

1.1 Spatiotemporally Resolved HHG Spectroscopy Technique ... 3

1.2 Least-Squares Fitting ... 5

1.3 Power Scaling of the Wave Mixings ... 6

1.4 SiO_2 Sample Properties ... 7

1.5 Dependence of the Third Harmonic Spectrum on Crystal Orientations ... 7

1.6 Definition for extracting energy centroid and harmonic yields depth ... 8

2 Numerical Simulations ... 9

2.1 *Ab initio* Calculation of Material Properties ... 9

2.2 Semiconductor Bloch Equations ... 11

2.3 Spatially Resolved Wave Mixing and Far-field Propagation ... 15

2.4 Dependence of Third Harmonic Spectrum on Dephasing Time ... 16

3 Analytical Considerations ... 17

3.1 Adiabatic Perturbation Theory for Density Matrix ... 17

3.2 Estimation of Energy Shifts ... 19

3.3 Third order Susceptibility of the Field-dressed Exciton ... 22

4 Supplementary References ... 24

1 Experimental Methods, Data Processing, and Sample Properties

1.1 Spatiotemporally Resolved HHG Spectroscopy Technique

As depicted in Fig.1**a** in the main text and supplementary Fig.1**a**, we conducted an experimental configuration where an intense fundamental laser pulse (ω_1 = 800 nm, ~1.55 eV) intersected a weaker second-harmonic field (ω_2 = 400 nm, ~3.1eV) in a non-collinear manner with the sample. The fundamental ω_1 and its second harmonic ω_2 are then recombined using a dichroic mirror, where ω_2 is reflected and the beams are spaced vertically downside by 5 mm. A similar experimental arrangement has previously been discussed in the generation of ultrahigh wave-mixing signals in the gas phase. A high-power laser system, Ti: Sapphire near-infrared (NIR) amplifier (Coherent Legend), generated the pump pulses with a carrier wavelength of 800 nm, operating at a repetition rate of 10 kHz and delivering a total energy of 1 mJ. The pump and probe pulses are maintained in the same fixed P-polarization. The sample is a crystalline SiO_2 (α-quartz crystal, *z*-cut plane, [0001]). A high-precision rotational stage was employed to ensure precise alignment of the crystal sample orientation concerning the incident laser pulses. This allowed for meticulous control and adjustment of the crystal's orientation, optimizing the interaction between the laser pulses and the crystal lattice. The temporal profiles of the pump and probe pulses were determined using our home-made transient grating frequency-resolved optical gating (TG-FROG) setup, yielding pulse durations of 25 ± 3 fs (Supplementary Fig. 1**b** and 1**c**) and 28± 4 fs (Supplementary Fig. 1**d** and 1**e**), respectively. To concentrate the light pulses, a 25 cm focal length lens was utilized, resulting in a beam size of approximately 25-40 μm, depending on the aperture size. The peak intensities used in the experiment are from ~10^{11} to ~10^{13} W/cm^2. The HHG spectra were captured using an extreme ultraviolet (EUV) spectrometer equipped with a flat-field variable groove density grating and a CCD camera-coupled micro-channel plates (MCP) detector. Covering a spectral range of 6 eV to 35 eV, the spectrometer's resolution was approximately 0.1 eV. The non-collinear angle between the pump and probe pulses was estimated to be ~18 mrad, determined from the angles of the harmonics observed on the MCP detector. For delay scans, a linearly closed-loop piezo stage was employed to precisely tune one of the pump-probe arms.

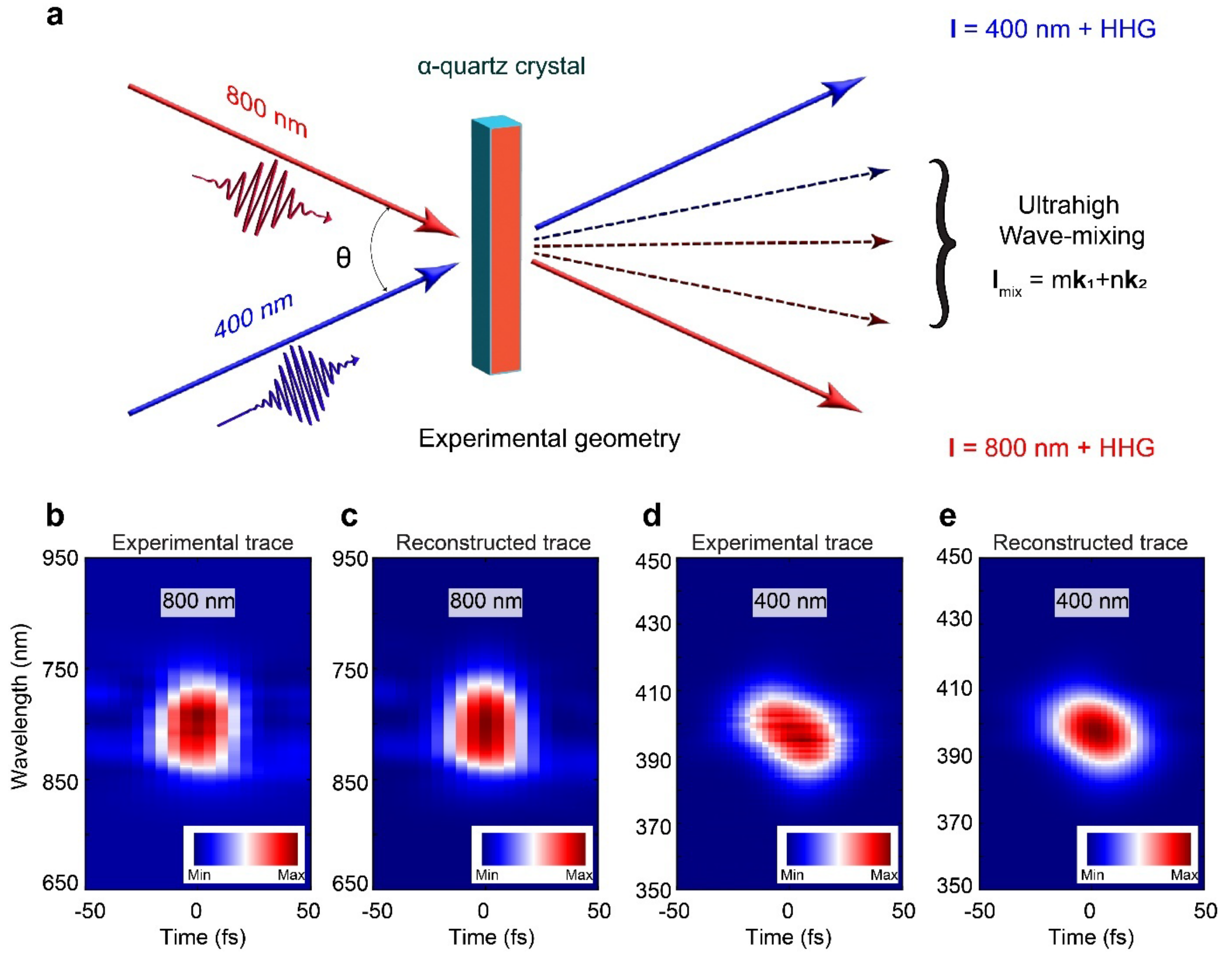

Supplementary Fig. 1 | Experimental geometry and pulse characterization. **a**, Schematic of the noncollinear geometry of the spatiotemporal resolved HHG measurement. Here, θ indicates the non-collinear angle between 800 nm pump and 400 nm probe pulses. The numbers m and n are integers, representing the number of absorbed photons from the pump and probe pulses, respectively. **b, d**, Measured and, **c**, **e**, reconstructed TG-FROG spectrum traces of 800 nm pump pulse, and 400 nm probe pulse, which gives a pulse duration of 25 ± 3 and 28 ± 4 fs, respectively. The color bars are normalized to their maximum harmonic yields and presented on a linear scale.

The noncolinear experimental configuration affords several advantages, including the ability to perform time-, energy-, momentum-, and parity-resolved HHG spectrum measurements. Furthermore, we also captured the time-resolved spectra of the pump field, as depicted in supplementary Figs. 2**a**-2**d**. These spectra encompass the harmonics ranging from the 5th to the 11th order when the intensity of the probe and pump pulse intensities set at 2×10^{11} W/cm^2 and 4×10^{12} W/cm^2, respectively. In this case, we observe an increase in the yields of the harmonics without any significant central energy shift. This behavior is distinct from the asymmetrical spectrum profiles exhibited by the harmonics generated from the probe field. The observed distinction between the weaker probe and the intensive

pump field's harmonics highlights field strength-dependent dynamics and responses of the system in the HHG process.

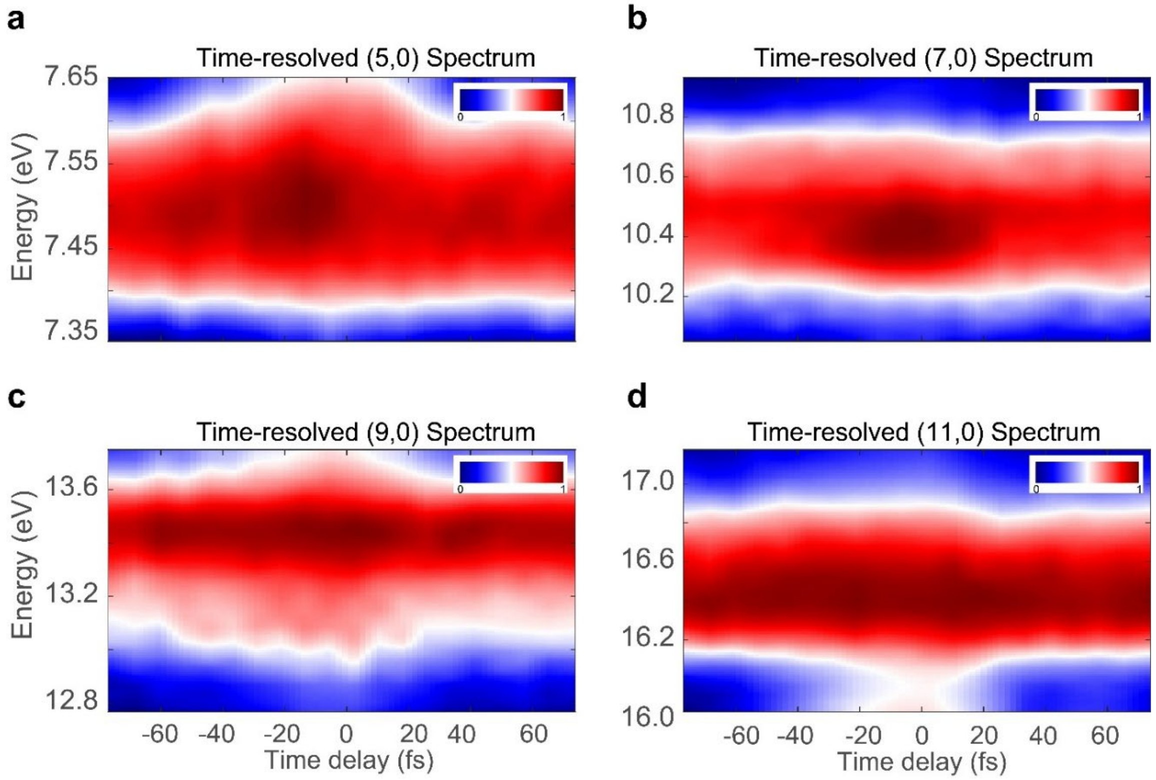

Supplementary Fig. 2 | Measured Time-Resolved HHG Spectra from the Pump Pulse. a to **d** The time-resolved spectra for (5, 0), (7, 0), (9, 0), and (11, 0) corresponding to the pump pulse are shown, with both pulses polarized in the Γ-K direction of the crystal. The color bars are normalized to the maximum of harmonic yields and presented on a linear scale.

1.2 Least-Squares Fitting

All the fittings performed in our study were carried out utilizing the Curve Fitting Toolbox in MATLAB software. This toolbox employs least-squares fitting methods to estimate the coefficients of a regression model. The algorithm used to calculate the vector of estimated responses is as follows

$$\hat{y} = f(X, b). \quad (1)$$

Here, $\hat{y}$ represents the response estimates, X is the design matrix, and b represents the parameters of the fitted model coefficients.

The least-squares fitting method aims to minimize the sum of squared errors (SSE), also known as the residual sum of squares. For a given set of n data points, the residual for the ith data point is calculated using the following equation

$$\mathrm{SSE} = \sum_{i=1}^{n} (y_i - \hat{y}_i)^2 \tag{2}$$

where y_i represents the observed response for the ith data point.

The fitting algorithm iteratively adjusts the coefficients b to minimize the SSE, resulting in the best fit of the regression model to the data.

1.3 Power Scaling of the Wave Mixings

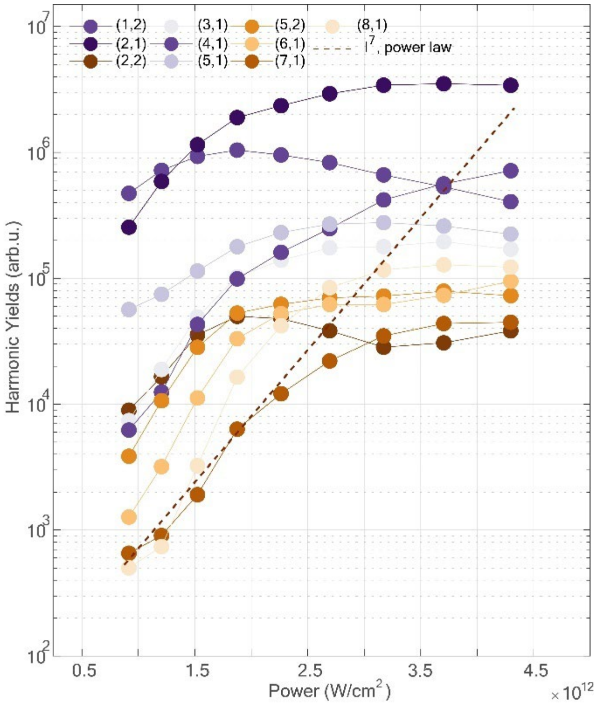

Supplementary Fig. 3 | Power-dependent scaling of the wave mixings. The recorded wave-mixing yields at varying laser intensities of the pump (ranging from ~0.9×10^{12} to 4.3×10^{12} W/cm^2) around zero delays are shown. The dashed line represents the power law fitting with I^7.

To confirm the excitation regime, we fixed the probe pulse intensity at 1×10^{12} W/cm^2 and the pump intensity-dependent yields of the wave-mixing spectra were analyzed, as shown in supplementary Fig. 3. It can be observed that, in the low intensity range the yields follow the perturbative I^n nonlinear dependence, while the power law fails to explain the observed tendency as the pump intensity increases, indicating that a nonperturbative regime at higher pump intensities is involved in the current experimental settings. In a noncollinear geometry, when examining the time-resolved high-harmonic generation spectra of the probe pulse, the pump pulse dynamically dresses the system,

including excitonic and Bloch states. In contrast, the probe pulse reflects the dynamics of carrier energy through its instantaneous dipole response.

1.4 SiO_2 Sample Properties

In our experiments, we employed a *z*-cut SiO_2 (α-quartz) crystal ([0001] orientation) provided by United Crystal, with dimensions of 5 × 5 mm, as the target for high-order harmonic generation. The crystal surface was optically polished on both sides, ensuring high-quality optical properties. To determine the crystal thickness, we utilized a custom-made white light interferometry spectrometer, which measured the thickness to be 20 ± 5 μm.

1.5 Dependence of the Third Harmonic Spectrum on Crystal Orientations

To investigate the impact of nonadiabatic energy shifts of electrons across these orientations, we first calculated the band structure of the quartz crystal using the density functional theory (DFT) and GW many-body perturbation theory (details provided in Section 2.1). Subsequently, we conducted experiments with the intensity of the pump and probe pulses maintained at 3.6×10^{12} W/cm² and 2×10^{11} W/cm², respectively. We recorded the time-resolved harmonic spectra while varying the crystal orientation in the Γ-M and Γ-K direction. The primary results of the time-dependent center of mass energy shift are presented in Supplementary Fig. 4**b**. At negative time delays, the energy shifts exhibit similar magnitudes, indicating that the redshift is comparable for both directions. At positive time delays, the blueshift for the Γ-K orientation are greater than that for the Γ-M orientation. This observation is consistent with lower effective mass and higher optical matrix elements along the Γ-K orientation compared to the Γ-M.

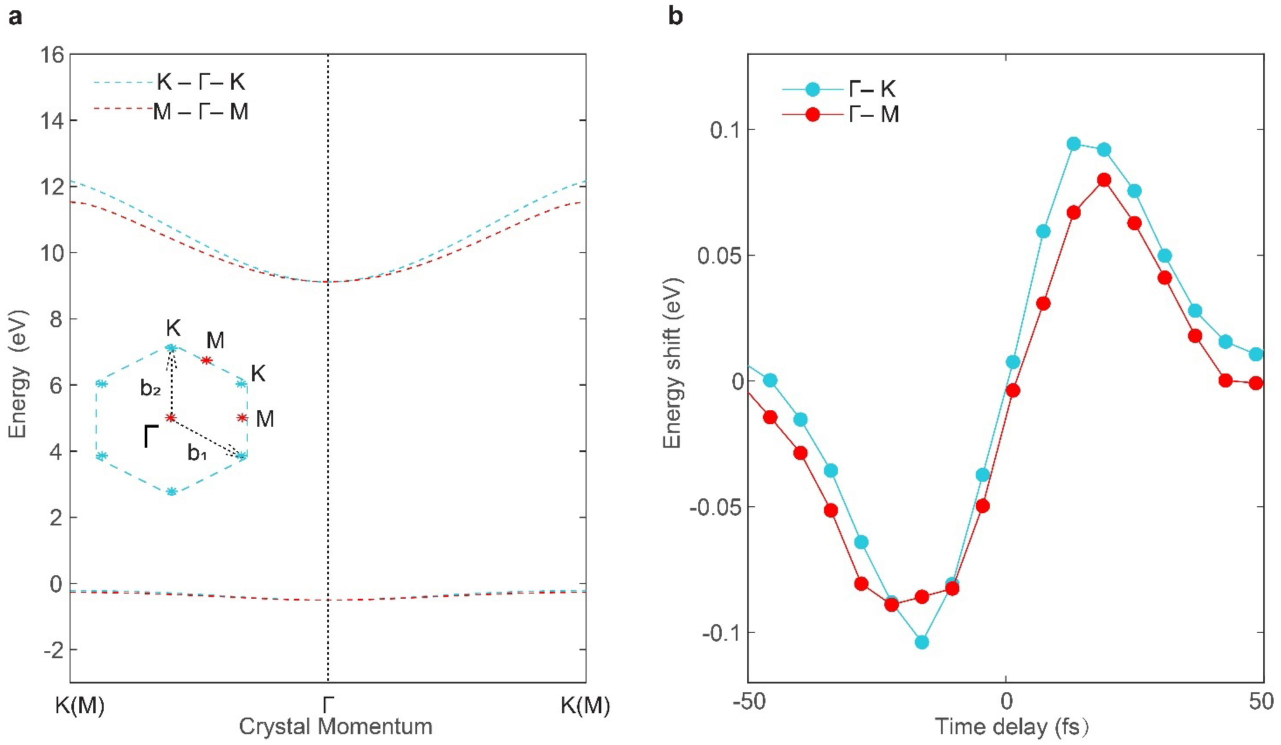

Supplementary Fig. 4 | Crystal orientation dependent tr-THG trace. a, Calculated band structure of α-quartz, highlighting one valence band and one conduction band for the Γ-M and Γ-K orientations. **b**, Experimentally extracted delay-dependent energy variation in the Γ-M (red) and Γ-K (blue) orientations.

1.6 Definition for extracting energy centroid and harmonic yields depth

To better understand our main figures (such as Fig. 2, Fig. 4, and Fig. 5) presented in the main text, we use the experimental data from Fig. 2 as an example to define the harmonic centroid energy, the bleaching effect, and their modulation depth, as illustrated in main Fig. 4. As shown in Supplementary Fig. 5**a**, we first select the H3 harmonic from the spatially resolved harmonic spectra (Fig. 1**b**) and plot it at different time delays. We then integrate the 3D data by summing it along the divergence angle direction to obtain a time-resolved spectrum, as shown in Supplementary Fig. 5**b**. For this 3D data, we perform the centroid analysis and energy domain analysis, resulting in Supplementary Figs. 5**c** and 5**d**, respectively. We can then extract the maximum harmonic yields and energy depth, as indicated by the double arrows.

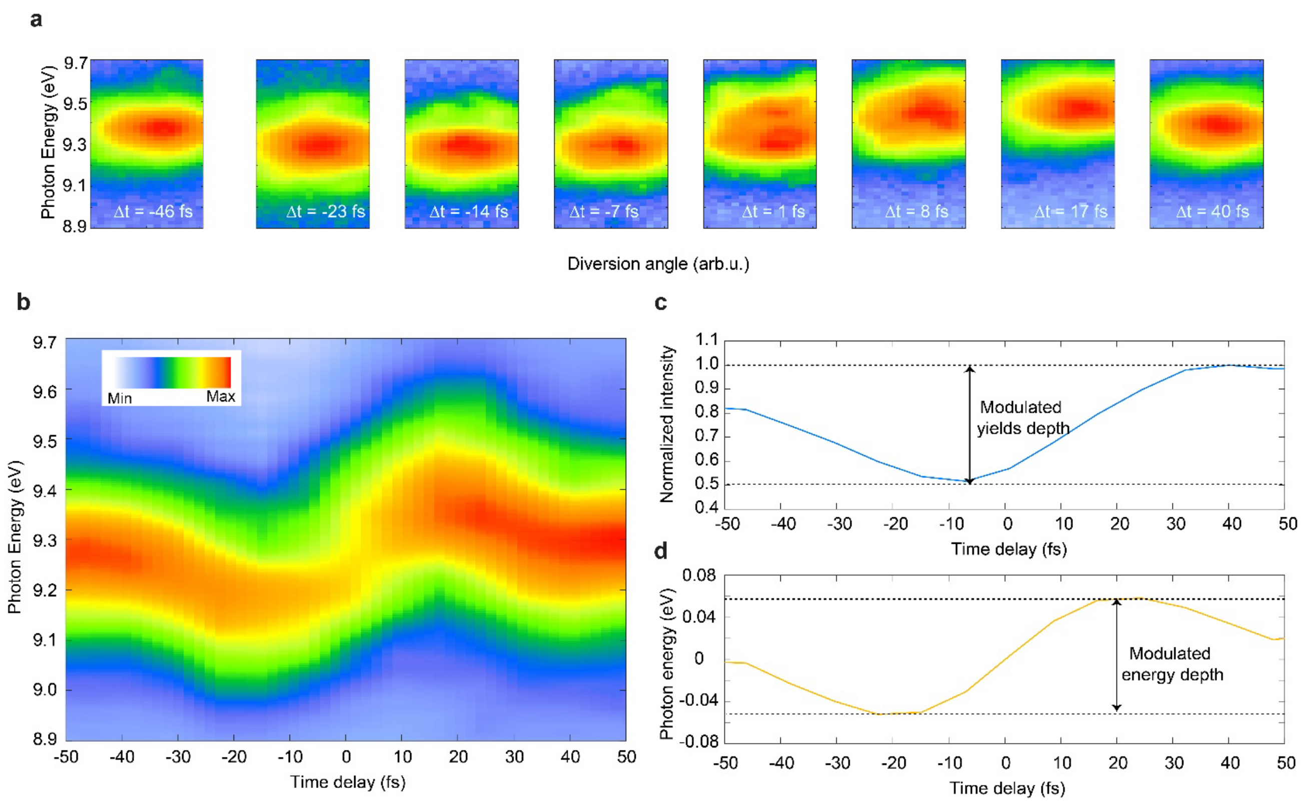

Supplementary Fig. 5 | Procedure for extracting the energy centroid and harmonic yields from time-resolved HHG measurements. a. THG spectra of the probe pulse at different time delays Δt. **b**. Integrated time-resolved THG trace for panel **a**, summed over the dispersion angle axis. **c** and **d**, Extracted harmonic yields and harmonic centroid energy variation at different time delays for panel **b**. The double arrows indicate the extracted modulation yields and energy depths presented in Figures 4**e** and 4**f**, respectively.

2 Numerical Simulations

2.1 *Ab initio* Calculations of Material Properties

The crystalline structure of α-quartz is characterized by a continuous framework of Si-O tetrahedra, where each oxygen atom is shared by two tetrahedra (see the elementary cell in the inset of Supplementary Fig. 6). Using these atomic coordinates as input, we performed an *ab initio* simulation of the quasiparticle band structure of α-quartz with the VASP code [1,2] (see Supplementary Fig. 6). Initial DFT calculation was done with the PBEsol GGA density functional [3]. The cutoff energy for the plane wave basis was set to 500 eV. The Brillouin zone was sampled with the zero-centered 11×11×11 Monhkorst-Pack grid. To include the carrier-carrier interaction terms beyond the GGA

which are essential for obtaining a correct quasiparticle band gap $E_{\mathrm{g}}^{\mathrm{QP}}$, we applied partially self-consistent GW_0 many-body perturbation theory [4,5]. Finally, the quasiparticle band structure was interpolated with the Wannier90 code [6].

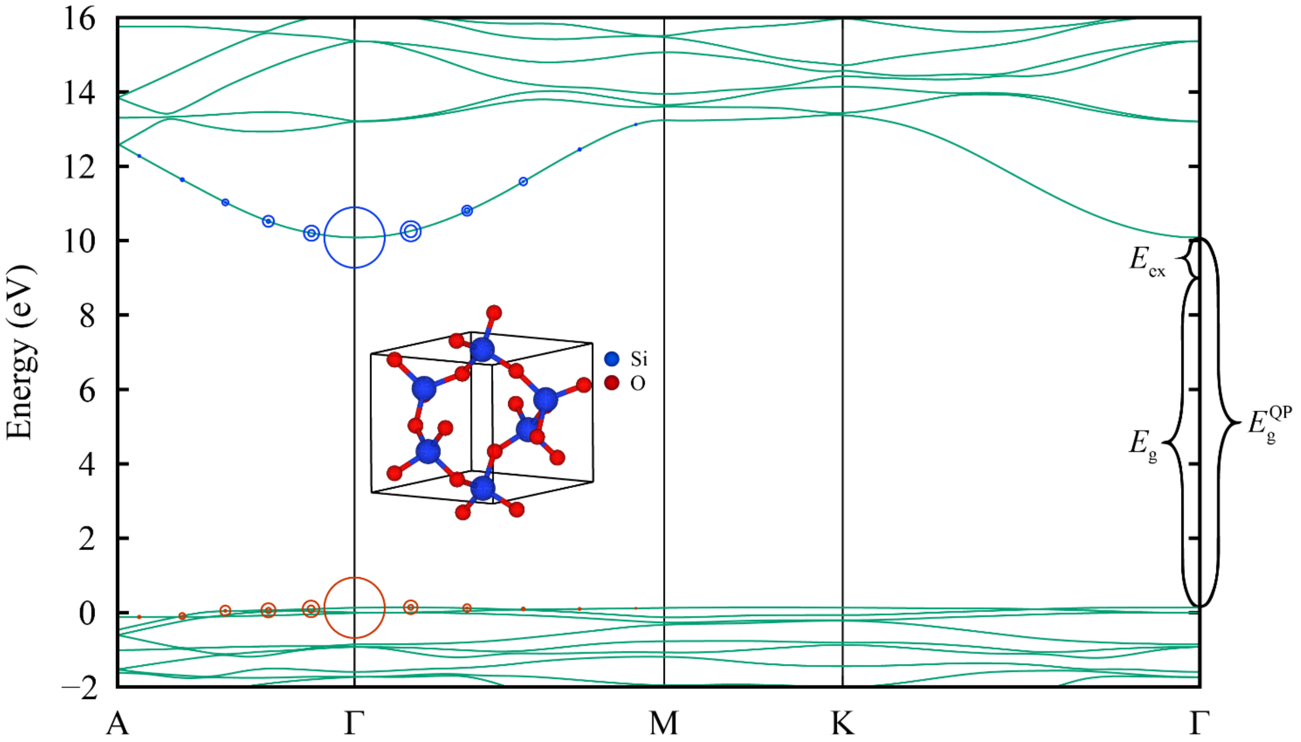

Supplementary Fig. 6 | Quasiparticle energy bands and the fat-band plot of α-quartz. Energy bands along the high-symmetry directions of the BZ are calculated with the PBEsol density functional and corrected by the GW_0 many-body perturbation theory. The radius of the circles is proportional to the absolute value of the coefficients $\left|\mathcal{A}_{\mathrm{cv}\mathbf{k}}^{(1A_1,0)}\right|$ describing the lowest excitonic $1A_1$ state as a superposition of the lowest conduction (c) and the highest valence (v) band states for two different spin orientations. Inset shows a unit cell of the α-quartz crystal, which is composed of the $(SiO_4)^{4-}$ anion complexes.

It is well-established that in the vicinity of the band gap, the optical properties of SiO_2 in both crystalline and fused silica forms are significantly influenced by carrier-carrier interactions [7–9]. They give rise to the formation of excitons, which could be described as a superposition of the Bloch state products

$$\left|s,\mathbf{Q}\right\rangle = \sum_{c,v,\mathbf{k}} \mathcal{A}_{cv\mathbf{k}}^{(s,\mathbf{Q})} a_{c\mathbf{k}+\mathbf{Q}}^{\dagger} a_{v\mathbf{k}} \left|0\right\rangle = \sum_{c,v,\mathbf{k}} \mathcal{A}_{cv\mathbf{k}}^{(s,\mathbf{Q})} \left|cv\mathbf{k}\right\rangle, \tag{3}$$

where $|0\rangle$ and $|s,\mathbf{Q}\rangle$ denote the vacuum and excited exciton states, respectively, $\mathbf{Q}$ is the exciton center-of-mass momentum, $a^{\dagger}_{c\mathbf{k}+\mathbf{Q}}$ and $a_{v\mathbf{k}}$ are creation and annihilation operators of electronic quasiparticles in the conduction c and valence v bands, respectively, with a crystal momentum $\mathbf{k}$ and exciton.

The coefficients $\mathcal{A}^{(s,\mathbf{Q})}_{cv\mathbf{k}}$ form an orthonormal set

$$\sum_{c,v,\mathbf{k}} \mathcal{A}^{(s,\mathbf{Q})*}_{cv\mathbf{k}} \mathcal{A}^{(s',\mathbf{Q})}_{cv\mathbf{k}} = \delta_{ss'}.$$

and satisfy the following secular equation for the poles of two-particle Green function known as the Bethe-Salpeter equation (BSE):

$$(E_{c\mathbf{k}+\mathbf{Q}} - E_{v\mathbf{k}})\mathcal{A}^{(s,\mathbf{Q})}_{cv\mathbf{k}} + \sum_{c',v',\mathbf{k}'} \mathcal{K}^{(s,\mathbf{Q})}_{cv\mathbf{k},c'v'\mathbf{k}'} \mathcal{A}^{(s,\mathbf{Q})}_{c'v'\mathbf{k}'} = \mathcal{E}_{s,\mathbf{Q}} \mathcal{A}^{(s,\mathbf{Q})}_{cv\mathbf{k}}, \tag{4}$$

where $E_{c\mathbf{k}}$ and $E_{v\mathbf{k}}$ are the quasiparticle band energies, $\mathcal{E}_{s,\mathbf{Q}}$ is the energy of excitonic state, and $\mathcal{K}^{(s,\mathbf{Q})}_{cv\mathbf{k},c'v'\mathbf{k}'}$ is the interaction kernel describing the scattering processes of an electron-hole pair from the state $|cv,\mathbf{k}\rangle$ to the state $|c'v',\mathbf{k}'\rangle$ on the Coulomb potential, emerging the excitonic state $|s,\mathbf{Q}\rangle$.

Given the matrix elements of an operator in the Bloch basis $O_{cv\mathbf{k}}$, such as band population or dipole of optical transition, the matrix elements in the exciton basis can be obtained as follows

$$O_{ss',\mathbf{Q}} = \sum_{c,v,\mathbf{k}} \mathcal{A}^{(s,\mathbf{Q})*}_{cv\mathbf{k}} O_{cv\mathbf{k}} \mathcal{A}^{(s',\mathbf{Q})}_{cv\mathbf{k}}.$$

To find an expansion of the excitonic state $1A_1$, we numerically solved the Bethe-Salpeter equation (4) with the VASP code and plotted the relative contributions of the electron and hole quasiparticle states at different $\mathbf{k}$ points $\left|\mathcal{A}^{(1A_1,\mathbf{Q})}_{cv\mathbf{k}}\right|$ with the circles of various radii in Supplementary Fig. 6 for two spin orientations.

2.2 Semiconductor Bloch Equations

To simulate the non-equilibrium dynamics, we solved the system of semiconductor Bloch equations in the time-dependent Hartree-Fock (TDHF) approximation

$$\begin{aligned} \partial_t \rho_{ij,\mathbf{k}}(t) = &-\mathrm{i}\left[\widehat{E}_{i,\mathbf{k}}(t) - \widehat{E}_{j,\mathbf{k}}(t) - \mathrm{i}\gamma_{ij}(t)\right]\rho_{ij,\mathbf{k}}(t) + \mathbf{F}(t)\cdot\partial_{\mathbf{k}}\rho_{ij,\mathbf{k}}(t) \\ &-\mathrm{i}\sum_l \left[\widehat{\Omega}_{il,\mathbf{k}}(t)\rho_{lj,\mathbf{k}}(t) - \rho_{il,\mathbf{k}}(t)\widehat{\Omega}_{lj,\mathbf{k}}(t)\right]. \end{aligned} \tag{5}$$

Here, we consider the laser field in the length gauge [10] and use the Crank-Nicolson method [11] for numerical integration.

In the TDHF approximation, carrier-carrier interaction leads to renormalization of energy bands

$$\widehat{E}_{i,\mathbf{k}}(t) = E_{i,\mathbf{k}} - \sum_{\mathbf{q}\neq\mathbf{k}} V_{|\mathbf{k}-\mathbf{q}|}\rho_{ii,\mathbf{q}}(t) \tag{6}$$

and interband interaction matrix elements (Rabi energies)

$$\begin{aligned} \widehat{\Omega}_{ij,\mathbf{k}}(t) &= \Omega_{ij,\mathbf{k}}(t) + \sum_{\mathbf{q}\neq\mathbf{k}} V_{|\mathbf{k}-\mathbf{q}|}\rho_{ij,\mathbf{q}}(t), \\ \Omega_{ij,\mathbf{k}}(t) &= \mathbf{F}(t)\cdot\mathbf{d}_{ij,\mathbf{k}} \end{aligned} \tag{7}$$

coupling the density matrix elements at various $\mathbf{k}$ points, $\mathbf{d}_{ij,\mathbf{k}} = \langle u_{i,\mathbf{k}} | \mathrm{i}\partial_{\mathbf{k}} | u_{j,\mathbf{k}} \rangle$ are the interband matrix elements of the coordinate operator in the crystal momentum representation, and

$$\gamma_{ij}(t) = (1-\delta_{ij})\gamma(t) \tag{8}$$

are the dephasing rates due to scattering on lattice defects, carrier-phonon, and many body interactions beyond the TDHF approximation.

Since the Coulomb correlations are mediated by microscopic polarizations, their impact depends on how fast these polarizations decay. It is shown that Coulomb correlations of the electron-hole pairs lead to a dramatic enhancement of HHG yields by the coupling of the interband polarizations to excitonic resonances with strong oscillator strengths, which open additional pathways for HHG [12,13].

We investigated how the excitonic many-body effect influences dynamic electronic energies when dressing with a strong field. The optical characteristics of α-quartz crystal are significantly influenced by excitonic effects, which renormalizes the quasiparticle band structure. In our 1D numerical SBEs simulation, we included electron-electron interaction in the time-dependent Hartree-Fock approximation using the soft-Coulomb potential [14]

$$V_q = \frac{V_0}{V}\int_V \mathrm{d}r \frac{\mathrm{e}^{iqr}}{\sqrt{r^2+1}} = V_0\frac{\Delta k}{\pi}K_0(q), \tag{9}$$

where k covers the BZ from $-\pi/a$ to π/a, lattice constant a = 9.45 a.u., N_k = 256, the crystal volume $V = 2\pi/\Delta k$ is estimated from the grid step size in the reciprocal space $\Delta k = 0.005/a$, $K_0(q)$ is the modified Bessel function of the second kind, and the interaction amplitude $V_0 = 0.52$ a.u. found by fitting the position of the first excitonic peak in the linear response spectrum calculated with the SBEs (5) to the experimental data [7,15] and *ab initio* solution of the BSE [16].

Response of a dielectric excited by a laser pulse naturally divides into the interband polarization density [10,17]

$$\mathbf{P}(t) = 2\sum_{i,j}\int_{\mathrm{BZ}} \frac{\mathrm{d}^3k}{(2\pi)^3}\rho_{ij,\mathbf{k}}(t)\mathbf{d}_{ij,\mathbf{k}} \tag{10}$$

and the intraband current density

$$\mathbf{J}(t) = 2\sum_i \int_{\mathrm{BZ}} \frac{\mathrm{d}^3 k}{(2\pi)^3} \rho_{ii,\mathbf{k}}(t) \partial_{\mathbf{k}} E_{i,\mathbf{k}} , \tag{11}$$

where the multiplier 2 accounts for spin degeneracy of Bloch states.

The total HHG emission spectrum is found as follows

$$I_{\mathrm{HHG}}(\omega) \propto \left| \omega \tilde{\mathbf{P}}(\omega) + \mathrm{i}\tilde{\mathbf{J}}(\omega) \right|^2 . \tag{12}$$

To evaluate static linear optical properties, we compute the electron dynamics under a weak field E(t) with a strength around $\sim 2\times10^5$ V/m, pulse duration of 40 fs, and center frequency at 400 nm. Furthermore, based on the weak-field-induced current response, the linear optical conductivity of quartz system can be evaluated as

$$\tilde{\sigma}(\omega) = \frac{\left| \omega \tilde{\mathbf{P}}(\omega) + \mathrm{i}\tilde{\mathbf{J}}(\omega) \right|^2}{\left| \tilde{\mathbf{F}}(\omega) \right|} , \tag{13}$$

where $\tilde{\mathbf{F}}(\omega)$ is the Fourier transform of the weak electric field $\mathbf{F}(t)$.

Supplementary Fig. 7 shows the dependence of linear conductivity spectra (**b**) and time-resolved THG spectra (**c**) on interaction amplitude V_0 varying from 0 to 0.63 a.u. leads to different excitonic spectra. The position of the first excitonic peak position is successfully reproduced at $V_0 = 0.52$ a.u.

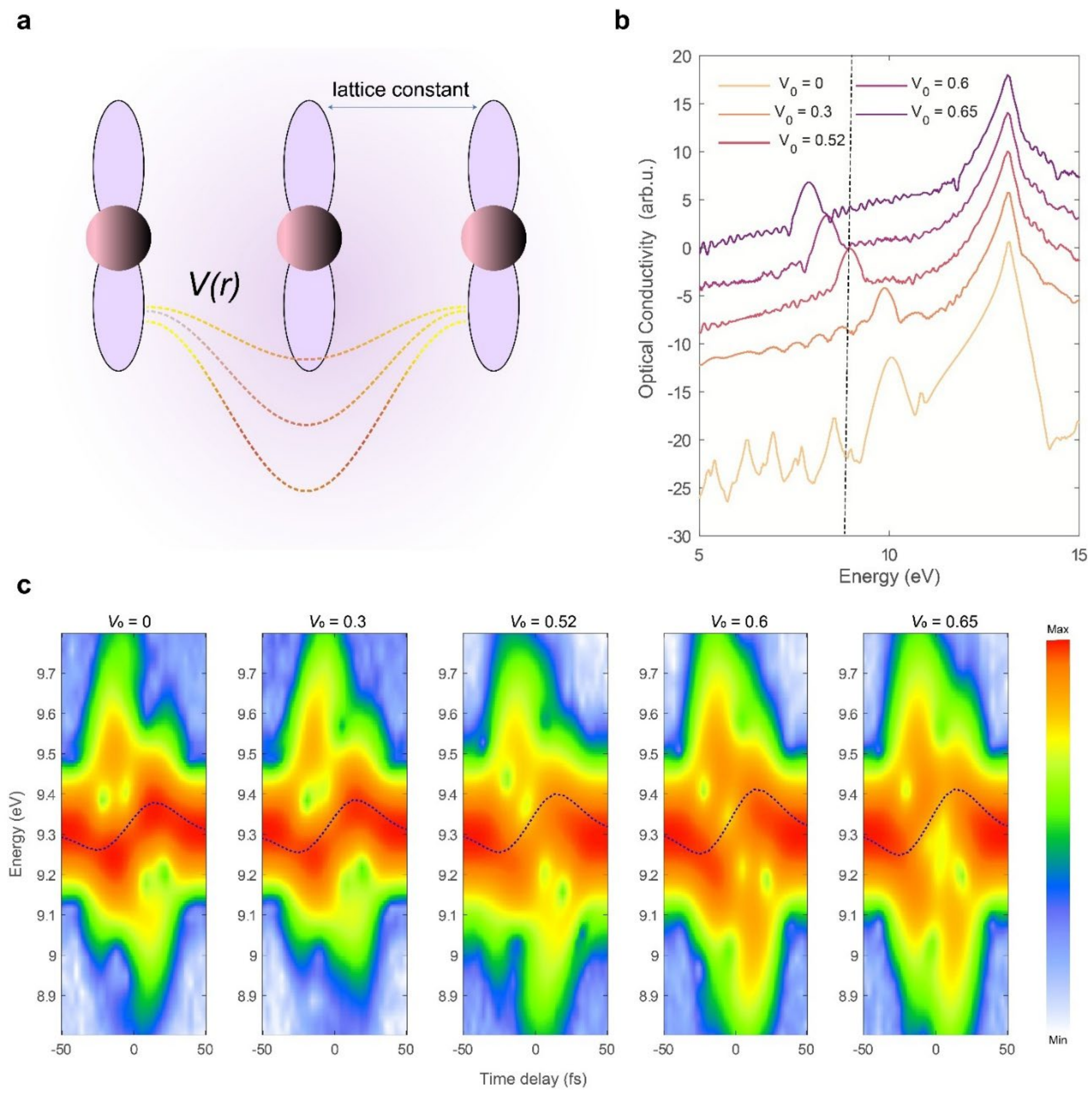

Supplementary Fig. 7 | Linear optical conductivity spectra calculated for different Coulomb interaction amplitudes. a, Schematic overview of Coulomb engineering for the band structure under different Coulomb interaction strengths $V(r)$ in 1D. **b,** Calculated real part of the optical conductivity varied at different Coulomb interaction amplitude V_0, the vertical dashed line indicates the position of the excitonic peak obtained from the *ab initio* solution of BSE. **c,** Influence of the different Coulomb interaction strengths on the time-resolved H3. The color bars are normalized to the maximum of harmonic yields and presented on a logarithmic scale. The dashed blue lines depict the centroid energy shift of the THG spectra.

Increasing the interaction energy of the charge carriers, as illustrated in Supplementary Fig. 8, leads to a larger modulation of the asymmetric spectrum, indicating that both the quadratic AC Stark

and ponderomotive effects become more pronounced. This effect arises because, under the influence of a light field, the strong electron-hole interaction weakens the pathway for interband tunneling.

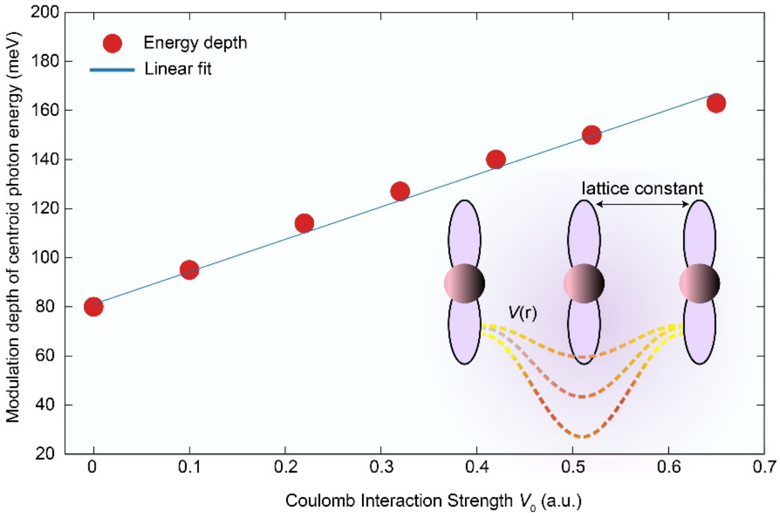

Supplementary Fig. 8 | Simulated carrier energy shift in the presence of different Coulomb potential amplitude. Variation of the extracted energy modulation depth (peak redshift to peak blueshift) the different Coulomb interaction amplitude V_0 (in a.u.) on the time-resolved H3, along with fits following the linear trend (solid blue line). The inset shows a one-dimensional schematic overview of carrier-carrier interactions by varying the Coulomb potential amplitude.

2.3 Spatially Resolved Wave Mixing and Far-field Propagation

To simulate the spatiotemporal resolved HHG spectra, we numerically solved above SBEs by setting up the total electric field of 400 nm and 800 nm in the time and real space as follows:

$$F(t)=\sum_{i=1,2}F_i\exp\left[\frac{t}{\tau_i}\right]^2\exp\left[\frac{x}{\mu_i}\right]^2\cos(\omega_i t-k_i x+\varphi_i), \tag{14}$$

where t and x are the time and space variables, τ_i and μ_i are the pulse duration and beam size at the focus point, F_i, ω_i, k_i, and φ_i are the amplitudes, carrier frequencies, wave vectors, and carrier-envelope phases of the laser pulses. In the simulation, the dephasing time T_2 is assumed to be 3 fs, and the grid step sizes for time and real space are 0.0049 mm and 0.048 fs, respectively, covering a range from -10 mm to 10 mm and -100 fs to 100 fs.

To simplify the simulation, we set $k_1=2\pi\sin\theta/\lambda_1$ and $k_2=0$, where θ is the noncolinear angle of the two pulses. To visualize the spatially resolved spectra, we first conducted a noncollinear near-field simulation using the SBEs in Eq. (5). We then propagate $I_{\mathrm{HHG}}(\omega,x)$ in free space around 1 m as shown in Fig. 1**d** and 1**e** in the main text.

2.4 Dependence of Third Harmonic Spectrum on Dephasing Time

Supplementary Fig. 9 shows how the calculated third harmonic spectrum changes with the dephasing time. For longer dephasing times, we observe rich interference structure with multiple side-lobes. This structure vanishes with a decrease of T_2 and the spectrum features the Gaussian-like distribution, a signature of a semiclassical particle evolution. The center-of-mass energy shift persists even at an extremely short dephasing time of 0.5 fs, which confirms its population dependence and robustness to decoherence.

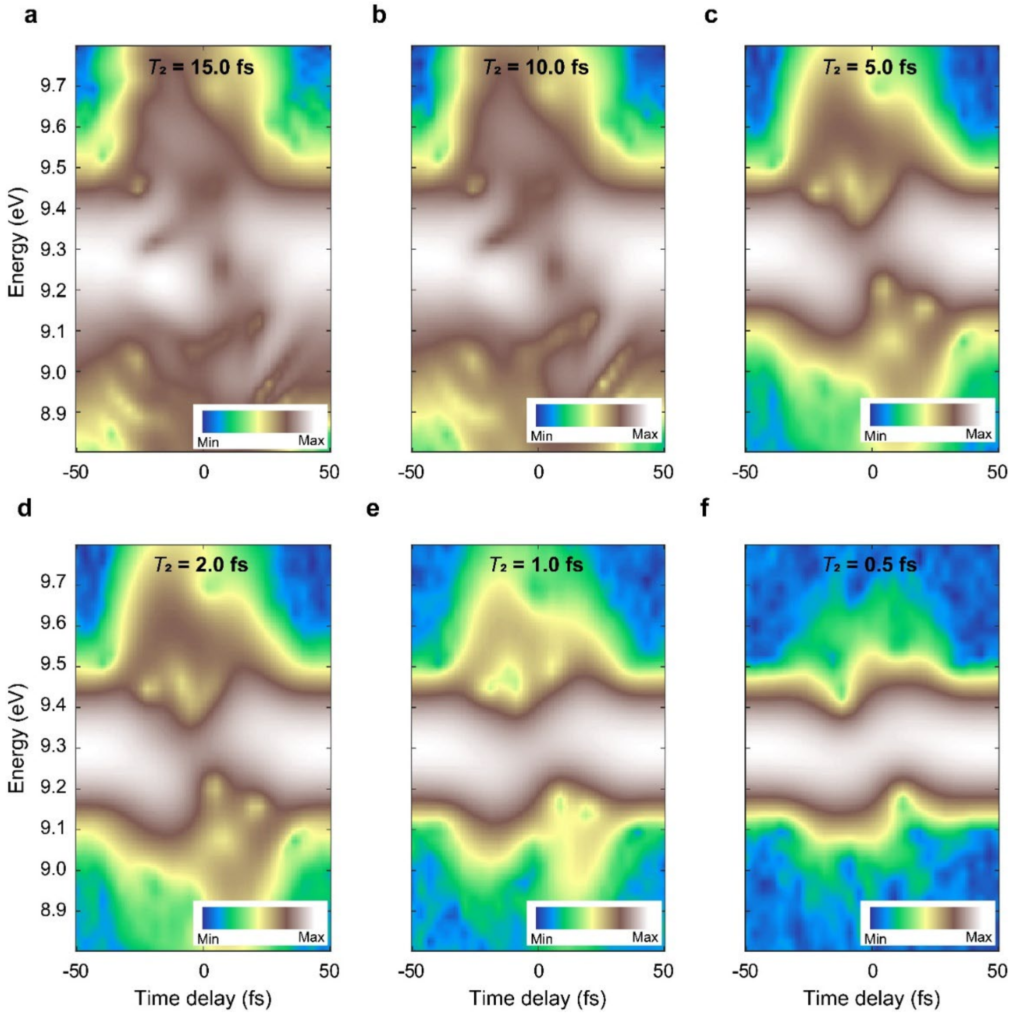

Supplementary Fig. 9 | Variation of time-resolved THG spectra under different dephasing times. Panels (**a-f**) depict the spectra corresponding to the dephasing time T_2 changing from 15 fs to 0.5 fs (bottom). The color bars are normalized and presented on a logarithmic scale. The laser intensity used for the pump and probe pulses is 5×10^{12} and 2×10^{11} W/cm^2, respectively.

3 Analytical Considerations

3.1 Adiabatic Perturbation Theory for Density Matrix

For analytical derivation of the field-induced energy shift, we start from the SBE for non-interacting quasiparticles and apply the method of characteristics to convert the system of partial differential equations (5) to the system of ordinary differential equations. This operation leads to the Peierls substitution and Houston basis. For brevity, we omit time argument of the time-dependent crystal momentum $\mathbf{K}(t) \to \mathbf{K}$.

To simplify further analysis, we neglect electron-electron interaction and use the diabatic interaction picture, where adiabatic part of the Hamiltonian $H'_{0,\mathbf{K}}(t)$ includes the lattice potential without the laser field H_0 and adiabatic parts of interaction with the field: 1) intraband motion described with the time-dependent crystal momentum $\mathbf{K}(t)$, and 2) adiabatic band shifts $\Omega_{ii,\mathbf{K}}(t) = \mathbf{F}(t) \cdot \mathbf{d}_{ii,\mathbf{K}}$ due to non-zero Berry connections, which could take place in materials with broken spatial inversion or time-reversal symmetry [18]. Operators this picture are connected with the operators in the Schrödinger picture via the transform $\tilde{O}(t) = U_0^\dagger O U_0$, where the evolution operator is given by

$$U_0(t,t_0) = \exp\left\{ -\mathrm{i}\int_{t_0}^{t} \mathrm{d}t_1\ H'_{0,\mathbf{K}}(t_1) \right\}. \tag{15}$$

The SBEs in the diabatic interaction picture can be written as

$$\frac{\mathrm{d}}{\mathrm{d}t}\tilde{\rho}_{ij,\mathbf{K}}(t) = -\mathrm{i}\sum_{l}\left[\tilde{\Omega}_{il,\mathbf{K}}(t)\tilde{\rho}_{lj,\mathbf{K}}(t) - \tilde{\rho}_{il,\mathbf{K}}(t)\tilde{\Omega}_{lj,\mathbf{K}}(t)\right] - \gamma_{ij,\mathbf{K}}(t)\tilde{\rho}_{ij,\mathbf{K}}(t), \tag{16}$$

where the light-matter interaction and density matrix elements are given by

$$\tilde{\Omega}_{ij,\mathbf{K}}(t) = \Omega_{ij,\mathbf{K}}(t)\exp\left\{ \mathrm{i}\int_{t_0}^{t} \mathrm{d}t_1\ \Delta E'_{ij,\mathbf{K}}(t_1) \right\}, \tag{17}$$

$$\tilde{\rho}_{ij,\mathbf{K}}(t) = \rho_{ij,\mathbf{K}}(t)\exp\left\{ \mathrm{i}\int_{t_0}^{t} \mathrm{d}t_1\ \Delta E'_{ij,\mathbf{K}}(t_1) \right\}. \tag{18}$$

Here, $\Delta E'_{ij,\mathbf{K}}(t) = E'_{i,\mathbf{K}} - E'_{j,\mathbf{K}}$, $E'_{i,\mathbf{K}}(t) = E_{i,\mathbf{K}} + \Omega_{ii,\mathbf{K}}(t)$.

The density matrix can be expressed in a series of powers of the non-diagonal (diabatic) part of interaction $\tilde{\Omega}_{ij,\mathbf{K}}(t)$:

$$\tilde{\rho}(t) = \tilde{\rho}^{(0)}(t) + \tilde{\rho}^{(1)}(t) + \tilde{\rho}^{(2)}(t) + \ldots \tag{19}$$

For the zeroth-order term, we have the initially populated valence bands

$$\tilde{\rho}_{ij}^{(0)}(t_0) = \begin{cases} \delta_{ij}, & i \in \mathrm{VB}; \\ 0, & \text{otherwise}. \end{cases} \tag{20}$$

For the first-order term, we have

$$\begin{aligned} \tilde{\rho}_{ij}^{(1)}(t) &= -\mathrm{i}\int_{t_0}^{t} \mathrm{d}t_1 \left[\tilde{\Omega}(t_1), \tilde{\rho}^{(0)}(t_0)\right]_{ij} \exp\left\{-\int_{t_0}^{t_1} \mathrm{d}t_2\, \gamma_{ij}(t_2)\right\} \\ &= \mathrm{i}\int_{t_0}^{t} \mathrm{d}t_1\, \widetilde{\Omega}_{ij}(t_1)\left[\tilde{\rho}_{ii}^{(0)}(t_1) - \tilde{\rho}_{jj}^{(0)}(t_0)\right] = \mathrm{i}\int_{t_0}^{t} \mathrm{d}t_1\, \widetilde{\Omega}_{ij}(t_1)\Delta n_{ij}^{(0)}, \end{aligned} \tag{21}$$

where the wide tilde over the matrix element means inclusion of the relaxation term into its phase multiplier

$$\widetilde{\Omega}_{ij,\mathbf{K}}(t_1) = \tilde{\Omega}_{ij,\mathbf{K}}(t_1)\exp\left\{-\int_{t_0}^{t_1} \mathrm{d}t_2\, \gamma(t_2)\right\} = \Omega_{ij,\mathbf{K}}(t_1)\exp\left\{\mathrm{i}\int_{t_0}^{t_1} \mathrm{d}t_2 \left[\Delta E'_{ij,\mathbf{K}}(t_2) + \mathrm{i}\gamma(t_2)\right]\right\}, \tag{22}$$

and $\Delta n_{ij}^{(0)} = \rho_{ii}^{(0)}(t_0) - \rho_{jj}^{(0)}(t_0)$ is the initial band population difference.

The second-order correction to density matrix is obtained from the first-order correction

$$\begin{aligned} \tilde{\rho}_{ij}^{(2)}(t) &= (-i)^2\int_{t_0}^{t} \mathrm{d}t_1 \left[\tilde{\Omega}(t_1), \tilde{\rho}^{(1)}(t_1)\right]_{ij} \exp\left\{-\int_{t_0}^{t_1} \mathrm{d}t_2\, \gamma_{ij}(t_2)\right\} \\ &= -\int_{t_0}^{t} \mathrm{d}t_1 \sum_{l}\left[\tilde{\Omega}_{il}(t_1)\tilde{\rho}_{lj}^{(1)}(t_1) - \tilde{\rho}_{il}^{(1)}(t_1)\tilde{\Omega}_{lj}(t_1)\right]\exp\left\{-\int_{t_0}^{t_1} \mathrm{d}t_3\, \gamma_{ij}(t_3)\right\} \\ &= \int_{t_0}^{t} \mathrm{d}t_1 \int_{t_0}^{t} \mathrm{d}t_2 \sum_{l}\left\{\mathrm{i}\widetilde{\Omega}_{il}(t_1)\widetilde{\Omega}_{lj}(t_2)\Delta n_{lj}^{(0)} - \mathrm{i}\widetilde{\Omega}_{il}(t_2)\widetilde{\Omega}_{lj}(t_1)\Delta n_{il}^{(0)}\right\}. \end{aligned} \tag{23}$$

The energy shift of electron wavepacket's center-of-mass is given by the second-order energy correction averaged by optical cycle and BZ

$$\Delta E_i^{(2)}(t) = \int_{t-T_{0,1}/2}^{t+T_{0,1}/2} \frac{\mathrm{d}t'}{T_{0,1}} \sum_{\mathbf{k},j}\left\{\rho_{ij,\mathbf{K}}^{(1)}(t')\Omega_{ji}(t') + \rho_{ij,\mathbf{K}}^{(2)}(t')E_{j,\mathbf{K}}(t')\right\}. \tag{24}$$

When the probe pulse is much weaker and shorter than the pump pulse, it can be approximated by the Dirac delta $\delta(t-\Delta t)$ and assume that the carriers are only excited by the pump pulse. Thus, the second-order energy correction for the energy of electron-hole pair can be approximated by the following expression:

$$\Delta E_i^{(2)}(\Delta t) \approx \sum_{\mathbf{k},j\neq i}\left|\Omega_{ij,\mathbf{K}_1}^{(0,1)}(\Delta t)\right|^2 \mathrm{Re}\left[\frac{\Delta\overline{n}_{ij,\mathbf{K}_1}(\Delta t)}{\Delta\overline{E}'_{ij,\mathbf{K}_1}(\Delta t) + \mathrm{i}\overline{\gamma}_{ij,\mathbf{K}_1}(\Delta t)}\right], \tag{25}$$

where overline symbol means cycle averaging, $\Omega^{(0,1)}_{ij,\mathbf{K}_1}(\Delta t) = \mathbf{F}_{0,1}(\Delta t)\cdot \overline{\mathbf{d}}_{ij,\mathbf{K}_1}(\Delta t)$ is the envelope Rabi energy, $\Delta\overline{n}_{ij,\mathbf{K}_1}(\Delta t) = \overline{\rho}_{ii,\mathbf{K}_1}(\Delta t) - \overline{\rho}_{jj,\mathbf{K}_1}(\Delta t)$ is the difference between cycle-averaged band populations in the electronic representation, $\mathbf{F}_{0,1}(\Delta t)$ is the pump pulse envelope multiplied by the unit vector in its polarization direction, $\Delta\overline{E}_{ij,\mathbf{K}_1}(\Delta t) = \overline{E}_{i,\mathbf{K}_1}(\Delta t) - \overline{E}_{j,\mathbf{K}_1}(\Delta t)$ is the cycle-averaged instantaneous energy of the electron-hole pair driven by the pump field. If the Berry connections are negligible, one can replace $\Delta\overline{E}'_{ij,\mathbf{K}_1}(\Delta t)$ with $\Delta\overline{E}_{ij,\mathbf{K}_1}(\Delta t)$.

This result can be further generalized to the case of excitonic states by expressing them as superpositions of electron-hole pair states (3) resulting in the expression

$$\Delta\mathcal{E}^{(2)}_i(\Delta t) \approx \sum_{\mathbf{k},j\neq i} \left|\Omega^{(0,1)}_{ij,\mathbf{K}_1}(\Delta t)\right|^2 \mathrm{Re}\left[\frac{\Delta\overline{n}_{ij,\mathbf{K}_1}(\Delta t)}{\Delta\overline{\mathcal{E}}_{ij,\mathbf{K}_1}(\Delta t) + \mathrm{i}\gamma}\right], \tag{26}$$

which is similar to (25), where the band energies are replaced by the exciton energies $\Delta\mathcal{E}'_{ij,\mathbf{K}_1}(\Delta t)$, the dipole matrix elements between Bloch bands are replaced with the matrix elements between the excitonic states $\mathbf{d}_{ij,\mathbf{K}_1}(\Delta t) = \sum_{c,v,\mathbf{k}'} \mathcal{A}^{(i,\mathbf{K}_1)*}_{cv,\mathbf{k}'} \mathcal{A}^{(j,\mathbf{K}_1)}_{cv,\mathbf{k}'} \mathbf{d}_{cv,\mathbf{k}'}$, $\Delta\overline{n}_{ij,\mathbf{K}_1}(\Delta t) = \overline{n}_{i,\mathbf{K}_1}(\Delta t) - \overline{n}_{j,\mathbf{K}_1}(\Delta t)$ is the difference between the cycle-averaged exciton level populations, which can be found by transforming the conduction and valence band populations in the electronic representation as

$$n_{i,\mathbf{K}_1}(\Delta t) = \sum_{c,v,\mathbf{k}'} \left|\mathcal{A}^{(i,\mathbf{K}_1)}_{cv\mathbf{k}'}\right|^2 n_{c,\mathbf{k}'+\mathbf{K}_1}(\Delta t)\left[1 - n_{v,\mathbf{k}'}(\Delta t)\right],$$

$\mathcal{A}^{(i,\mathbf{K}_1)}_{cv\mathbf{k}}$ are the coefficients of the exciton state expansion obtained via solution of the Bethe-Salpeter equation (4).

3.2 Estimation of Energy Shifts

For simplicity, we assume that the charge carriers [19]are mainly excited by the pump pulse and neglect the strong-field effects, e.g., intraband acceleration and energy renormalization, induced by the probe pulse. In the picture given by the TDHF Eq. (5), Coulomb interaction dynamically renormalizes the band energies, introduces excitonic states shifted from the quasiparticle bandgap by the exciton binding energy $E_\mathrm{g} = E^\mathrm{QP}_\mathrm{g} - E_\mathrm{ex}$. This effect significantly changes the carrier excitation process, especially if the exciton binding energy is large, which is the case for α-quartz.

At low intensities, charge carriers are predominantly transferred to the excitonic states. Thus, before the interband transition due to the pump pulse we have the red excitonic Stark shift. After the

excitation, the charge carriers may experience blue shifts due to field-driven acceleration of excitonic states, their ionization, and acceleration of the Bloch states.

Substituting the excitonic states to the Eq. (26), we obtain the following expressions for the center-of-mass energy shift of the exciton as a function of delay Δt between the pump and probe pulses

$$\Delta\mathcal{E}(\Delta t) \approx \Delta\mathcal{E}^{(+)}(\Delta t) + \Delta\mathcal{E}^{(-)}(\Delta t). \tag{27}$$

Here, the first term

$$\begin{aligned}\Delta\mathcal{E}^{(+)}(\Delta t) = \int_{\mathrm{BZ}} \frac{\mathrm{d}^3k}{(2\pi)^3} \Bigg\{ \left|\Omega^{(0,1)}_{1A_1 2T_2,\mathbf{K}_1}(\Delta t)\right|^2 \mathrm{Re}\left[\frac{\Delta\overline{n}_{1A_1 2T_2,\mathbf{K}_1}(\Delta t)}{\Delta\overline{\mathcal{E}}_{1A_1 2T_2,\mathbf{K}_1}(\Delta t) + \mathrm{i}\gamma}\right] + \\ + \left|\Omega^{(0,1)}_{1A_1\,\mathrm{cv},\mathbf{K}_1}(\Delta t)\right|^2 \mathrm{Re}\left[\frac{\Delta\overline{n}_{1A_1\,\mathrm{cv},\mathbf{K}_1}(\Delta t)}{\Delta\overline{\mathcal{E}}_{1A_1\,\mathrm{cv},\mathbf{K}_1}(\Delta t) + \mathrm{i}\gamma}\right]\Bigg\}\end{aligned} \tag{28}$$

describes the blue shifts due to field-induced motion of the total exciton energy due to virtual transitions from the lowest excitonic $1A_1$ state to higher excitonic states and to the Bloch states, $\mathbf{K}_1$ is the kinetic crystal momentum of the exciton CoM motion in the pump field, $\mathbf{F}_{0,1}(\Delta t)$ is the pump pulse envelope multiplied by the unit vector in the field polarization direction.

The second term describes the redshift due to the quadratic Stark effect, which occurs during negative delays when the carriers are excited from the ground state

$$\Delta\mathcal{E}^{(-)}_{1A_1}(\Delta t) = \int_{\mathrm{BZ}} \frac{\mathrm{d}^3k}{(2\pi)^3} \left|\Omega^{(0,1)}_{1A_1 0,\mathbf{K}_1}(\Delta t)\right|^2 \mathrm{Re}\left[\frac{\Delta\overline{n}_{1A_1 0,\mathbf{K}_1}(\Delta t)}{\Delta\overline{\mathcal{E}}_{1A_1\,0,\mathbf{K}_1}(\Delta t) + \mathrm{i}\gamma}\right]. \tag{29}$$

For evaluation of our analytical model, we use the simple 1D model and the basis of two Bloch bands and $1A_1$ excitonic state. For simplicity, we neglect virtual transitions to and from the $2T_2$ state, so the major contributions to energy correction are given by

$$\Delta\mathcal{E}^{(+)}_{1A_1}(\Delta t) \approx F^2_{0,1}(\Delta t)\frac{E_{\mathrm{ex}}}{E^2_{\mathrm{ex}} + \gamma^2} \int_{\mathrm{BZ}} \frac{\mathrm{d}^3k}{(2\pi)^3} \left|\overline{\mathbf{d}}_{\mathrm{cv}\,1A_1,\mathbf{K}_1}(\Delta t)\right|^2 \Delta\overline{n}_{\mathrm{cv}\,1A_1,\mathbf{K}_1}(\Delta t), \tag{30}$$

$$\Delta\mathcal{E}^{(-)}_{1A_1}(\Delta t) \approx F^2_{0,1}(\Delta t)\frac{E_{\mathrm{g}}}{E^2_{\mathrm{g}} + \gamma^2} \int_{\mathrm{BZ}} \frac{\mathrm{d}^3k}{(2\pi)^3} \left|\overline{\mathbf{d}}_{1A_1 0,\mathbf{K}_1}(\Delta t)\right|^2 \Delta\overline{n}_{1A_1 0,\mathbf{K}_1}(\Delta t). \tag{31}$$

Time-dependent excitation probability $n_{\mathrm{c},\mathbf{K}_1}(t) \equiv \rho_{\mathrm{cc},\mathbf{K}_1}(t)$ can be estimated from the two-band approximation

$$\rho_{\mathrm{cc},\mathbf{K}}(t) = \mathrm{i}\int_{t_0}^{t} \mathrm{d}t_1\, \Omega_{\mathrm{cv},\mathbf{K}}(t_1)\rho_{\mathrm{cv},\mathbf{K}}(t_1)\mathrm{e}^{\mathrm{i}S_{\mathrm{cv},\mathbf{K}}(t_1)} + \mathrm{c.c.}, \tag{32}$$

where

$$S_{\mathrm{cv},\mathbf{K}}(t_1) = \int_{t_0}^{t} \mathrm{d}t_1\ \Delta E_{\mathrm{cv},\mathbf{K}}(t_1). \tag{33}$$

We neglect quantum interference effects and describe the excitation probability with the instantaneous rate of the 3-photon process for the pump pulse. This leads to the following approximate expressions:

$$n_{\mathrm{c}}(t) = \int_{\mathrm{BZ}} \mathrm{d}^3k\ \rho_{\mathrm{cc},\mathbf{K}}(t) \approx n_{\mathrm{c}}(t) \approx \gamma_{\mathrm{RF}}^{6}\Phi^{(6)}(t,T), \tag{34}$$

$$\gamma_{\mathrm{RF}} = \frac{\mathbf{F}_0 \cdot \mathbf{D}_{\mathrm{cv}}}{E_{\mathrm{g}}}, \quad \mathbf{D}_{\mathrm{cv}} = \int_{\mathrm{BZ}} \frac{\mathrm{d}^3k}{(2\pi)^3}\,\mathbf{d}_{\mathrm{cv}}(\mathbf{k}), \tag{35}$$

$$\Phi^{(6)}(t,T) = \int_{t_0}^{t} \mathrm{d}t_1\ f^{6}(t_1,T). \tag{36}$$

Here, γ_{RF} is the small parameter of the perturbation theory for the pump pulse, $\mathbf{D}_{\mathrm{cv}}$ is the averaged dipole matrix element, T is the pulse FWHM.

For the Gaussian pulse with an envelope given by

$$f(t,T) = \exp\left[-\frac{t^2}{2\sigma^2}\right], \tag{37}$$

we obtain

$$\Phi^{(N)}(t,T) = \sqrt{\frac{\pi}{2N}}\sigma\,\mathrm{erfc}\left[-\sqrt{\frac{N}{2}}\frac{t}{\sigma}\right], \tag{38}$$

where the pulse standard deviation σ relates to its FWHM as $\sigma = T/2\sqrt{\ln 2}$, and $\mathrm{erfc}(x)$ is the complementary error function.

For the peak pump pulse intensity of $F_{0,1} = 4.2\times10^{12} W/cm^2$ we obtained the result shown below in Supplementary Fig. 10**a**. In addition, scaling of the energy shift peak with the pump field amplitude is shown in the Supplementary Fig. 10**b**. The calculated values exhibit quadratic dependence on $F_{0,1}$ and diverge more significantly at higher pump field amplitudes due to difference in the transition matrix elements and level populations.

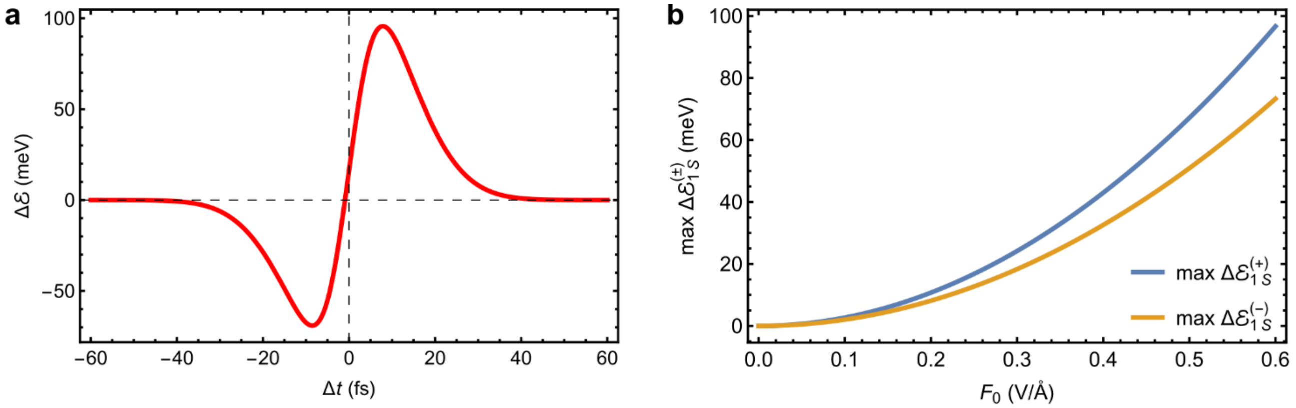

Supplementary Fig. 10 | Analytical results for the carrier energy shifts. a, Time delay-dependent energy shift estimated from the analytical theory. **b,** Peak energy shift amplitudes versus the pump field amplitude

3.3 Third order Susceptibility of the Field-dressed Exciton

Within the two-time method of quantum optics, where $T = \Delta t$ is a time of slowly-changing variables connected with the envelope of the pump pulse and τ is a time of fast-changing variables due to probe dynamics, we can introduce the generalized Floquet expansion of the excitonic states renormalized by the pump pulse and probed by the weak probe pulse

$$\Psi(\mathbf{r},T,\tau) = \sum_{\alpha\in\mathrm{TBZ}} c_\alpha(T)\phi_\alpha(\mathbf{r},T,\tau)\exp[-\mathrm{i}\tilde{\varepsilon}_\alpha(T)\tau], \tag{39}$$

$$\phi_\alpha(\mathbf{r},T,\tau) = \sum_n \phi_{\alpha,n}(\mathbf{r},T)\exp[-\mathrm{i}n\omega_2\tau], \tag{40}$$

where ω_2 is the central frequency of the probe pulse.

Substituting (40) to (39), we have

$$\Psi(\mathbf{r},T,\tau) = \sum_{\alpha\in\mathrm{TBZ}} \sum_n c_\alpha(T)\phi_{\alpha,n}(\mathbf{r},T)\exp\left\{-\mathrm{i}\left[\tilde{\varepsilon}_\alpha(T) + n\omega_2\right]\tau\right\},$$

where $\phi_\alpha(\mathbf{r},T,\tau)$ are the quasistatic Floquet states satisfying the quasi-stationary Schrödinger equation

$$\left[H_0(\mathbf{r}) + V_1(T) - \mathrm{i}\partial_T\right]\phi_\alpha(\mathbf{r},T,\tau) = \tilde{\varepsilon}_\alpha(T)\phi_\alpha(\mathbf{r},T,\tau). \tag{41}$$

Applying the perturbation theory in the Floquet basis and following the steps described in Refs. [20,21], we obtain the following expression of the retarded susceptibility describing the third-order response to the probe pulse from the excitonic states

$$\tilde{\chi}_i^{(3)}(T=\Delta t, 3\omega_2) \approx \sum_{\alpha,N}\sum_{n,l,m} d_{il}^{(\alpha)} d_{ln}^{(\alpha)} d_{nm}^{(\alpha)} d_{mi}^{(\alpha)}$$
$$\times\left\{\frac{1}{\left[\tilde{\Sigma}_{l,N}(\Delta t)-3\omega_2\right]\left[\tilde{\Sigma}_{n,N}(\Delta t)-2\omega_2\right]\left[\tilde{\Sigma}_{m,N}(\Delta t)-\omega_2\right]}\right.$$
$$+\frac{1}{\left[\tilde{\Sigma}_{l,N}(\Delta t)+\omega_2\right]\left[\tilde{\Sigma}_{n,N}(\Delta t)-2\omega_2\right]\left[\tilde{\Sigma}_{m,N}(\Delta t)-\omega_2\right]} \tag{42}$$
$$+\frac{1}{\left[\tilde{\Sigma}_{l,N}(\Delta t)+\omega_2\right]\left[\tilde{\Sigma}_{n,N}(\Delta t)+2\omega_2\right]\left[\tilde{\Sigma}_{m,N}(\Delta t)-\omega_2\right]}$$
$$\left.+\frac{1}{\left[\tilde{\Sigma}_{l,N}(\Delta t)+\omega_2\right]\left[\tilde{\Sigma}_{n,N}(\Delta t)+2\omega_2\right]\left[\tilde{\Sigma}_{m,N}(\Delta t)+3\omega_2\right]}\right\}.$$

Here,

$$\tilde{\Sigma}_{\alpha,N}(\Delta t) = \tilde{\varepsilon}_\alpha(\Delta t) + N\omega_2 + \mathrm{i}\tilde{\gamma}_\alpha(\Delta t) \tag{43}$$

are the self-energies of the Floquet resonances induced by excitonic states renormalized by the pump pulse, $\tilde{\gamma}_\alpha$ is the Floquet resonance damping rate, which could be time-dependent, in general, and $d_{nm}^{(\alpha)} \equiv d_{nm}^{(\alpha)}(\mathbf{r}=\mathbf{0})$ are the dipole matrix elements between the Floquet states, whose spatial dependency is neglected, as in Ref. [20].

Four terms in braces of Eq. (42) correspond to four different types of transitions contributing to the third-order response (see Supplementary Figs. 11**a** to 11**d**).

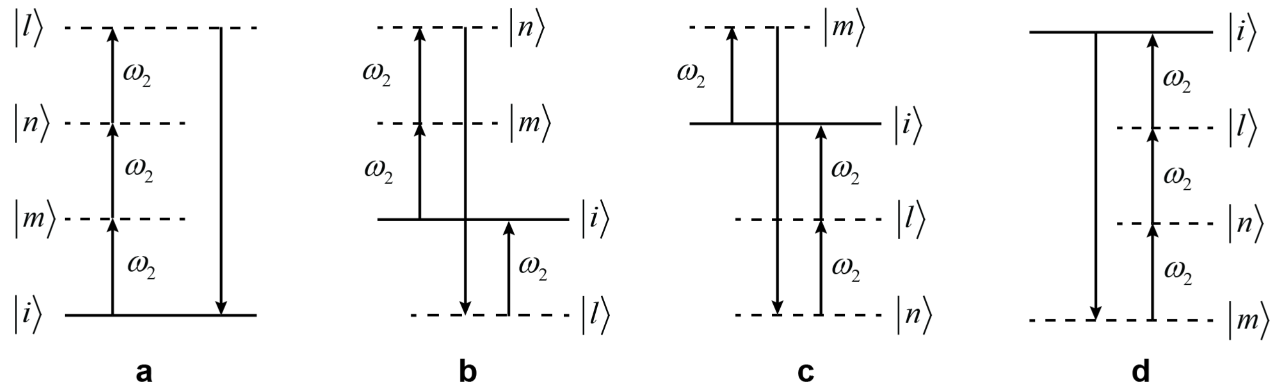

Supplementary Fig. 11 | Schematics of virtual transitions contributing to the third-order response function. a, Absorption of three photons from the probe pulse followed by emission of the third harmonic. **b,** Absorption of two photons followed by emission of the third harmonic and absorption of the third photon. **c,** Absorption of one photon followed by emission of the third harmonic and absorption of two photons. **d,** Emission of the third harmonic followed by absorption of three photons.

4 Supplementary References

[1] G. Kresse and J. Furthmüller, Efficient iterative schemes for ab initio total-energy calculations using a plane-wave basis set, Phys. Rev. B. **54**, 11169 (1996).

[2] G. Kresse, M. Marsman, L. E. Hintzsche, and E. Flage-Larsen, Optical and electronic properties of Si3N4 and α-SiO2, Phys. Rev. B. **85**, 045205 (2012).

[3] J. P. Perdew, A. Ruzsinszky, G. I. Csonka, O. A. Vydrov, G. E. Scuseria, L. A. Constantin, X. Zhou, and K. Burke, Restoring the Density-Gradient Expansion for Exchange in Solids and Surfaces, Phys. Rev. Lett. **100**, 136406 (2008).

[4] L. Hedin, New Method for Calculating the One-Particle Green's Function with Application to the Electron-Gas Problem, Phys. Rev. **139**, A796 (1965).

[5] M. Shishkin and G. Kresse, Implementation and performance of the frequency-dependent GW method within the PAW framework, Phys. Rev. B. **74**, 035101 (2006).

[6] G. Pizzi et al., Wannier90 as a community code: new features and applications, Journal of Physics: Condensed Matter **32**, 165902 (2020).

[7] H. R. Philipp, Optical transitions in crystalline and fused quartz, Solid State Commun **4**, 73 (1966).

[8] E. K. Chang, M. Rohlfing, and S. G. Louie, Excitons and optical properties of α-quartz, Phys. Rev. Lett. **85**, 2613 (2000).

[9] S. Ismail-Beigi and S. G. Louie, Self-trapped excitons in silicon dioxide: Mechanism and properties, Phys. Rev. Lett. **95**, 156401 (2005).

[10] O. Schubert et al., Sub-cycle control of terahertz high-harmonic generation by dynamical Bloch oscillations, Nat. Photonics **8**, 119 (2014).

[11] J. Crank and P. Nicolson, A practical method for numerical evaluation of solutions of partial differential equations of the heat-conduction type, Mathematical Proceedings of the Cambridge Philosophical Society **43**, 50 (1947).

[12] J. Hader, J. Neuhaus, J. V. Moloney, and S. W. Koch, Coulomb enhancement of high harmonic generation in monolayer transition metal dichalcogenides, Opt. Lett. **48**, 2094 (2023).

[13] E. B. Molinero, B. Amorim, M. Malakhov, G. Cistaro, Á. Jiménez-Galán, A. Picón, P. San-José, M. Ivanov, and R. E. F. Silva, Subcycle Dynamics of Excitons under Strong Laser Fields, 2024.

[14] Z. Yang, Y. Li, and C. A. Ullrich, A minimal model for excitons within time-dependent density-functional theory, J. Chem. Phys. **137**, 014513 (2012).

[15] H. R. Philipp, Silicon dioxide (SiO2) (glass), Handbook of Optical Constants of Solids **1**, 749 (2012).

[16] S. Ismail-Beigi and S. G. Louie, Self-trapped excitons in silicon dioxide: Mechanism and properties, Phys. Rev. Lett. **95**, 156401 (2005).

[17] D. Golde, T. Meier, and S. W. Koch, High harmonics generated in semiconductor nanostructures by the coupled dynamics of optical inter- and intraband excitations, Phys. Rev. B. **77**, 075330 (2008).

[18] T. Holder, D. Kaplan, and B. Yan, Consequences of time-reversal-symmetry breaking in the light-matter interaction: Berry curvature, quantum metric, and diabatic motion, Phys Rev Res **2**, 033100 (2020).

[19] C. Heide, P. D. Keathley, and M. F. Kling, Petahertz electronics, Nat. Rev. Phys. **6**, 648–662 (2024).

[20] R. Boyd, *Nonlinear Optics*, 4th ed. (Elsevier Science, London, 2020).

[21] H. Haug and A.-P. Jauho, *Quantum Kinetics in Transport and Optics of Semiconductors*, Vol. 123 (Springer Berlin Heidelberg, Berlin, Heidelberg, 2008).
